# Troponin Levels at Presentation Before Coronary Angiogram and Their Association With Cost, Mortality, and Readmission

**DOI:** 10.7759/cureus.12057

**Published:** 2020-12-13

**Authors:** Mansoor Ahmad, Nathan A Neilson, Jishanth Mattumpuram, Michael Tye, Muhammad Asghar, Minchul Kim, Sudhir Mungee

**Affiliations:** 1 Cardiology, University of Illinois Chicago, College of Medicine at Peoria, Peoria, USA; 2 Internal Medicine, University of Illinois Chicago, College of Medicine at Peoria, Peoria, USA

**Keywords:** cathpci, troponin level, cost, readmission, death

## Abstract

Background and objective

In patients undergoing coronary angiogram, the degree of cardiac enzyme elevation at presentation has been thought of as a strong and independent predictor of in-hospital mortality and readmission. Recent studies, however, have suggested a lack of clarity regarding this speculation, particularly with regard to troponin elevations. Additionally, the impact of troponin levels (TnI) at presentations on cost is poorly understood. In this study, we aimed to evaluate the association of Tnl at initial presentation with 30-day all-cause readmission and all-cause mortality as well as admission costs.

Methods

This study was a retrospective analysis of 7,388 patients who underwent coronary angiogram at our facility from 2015 to 2017. Patients were identified from a local CathPCI Registry^®^ registry, and a subsequent chart review was performed for readmission and mortality data. Cost data were provided by our in-facility finance department. We excluded patients with incomplete records and those who required coronary artery bypass grafting (CABG). After the exclusion of patients deemed ineligible, the final sample size eligible for analysis was 1,163. Patients were divided into two groups based on Tnl at presentation with a cut-off value of 0.04 ng/ml. Adjusted regression and multivariate analysis were used for clinical outcomes. Primary outcomes were 30-day readmission and mortality. The secondary outcome was the admission cost.

Results

Within our cohort, the average participant age was 64.6 years (SD: 12.7), and the majority of them were male (70.7%). Of these patients, 207 had lower TnI (<0.04 ng/ml), while 956 had higher TnI at presentation. The high TnI (≥0.04 ng/ml) group had a significantly higher number of patients with a family history of coronary artery disease (CAD) (13.8% vs. 7.7%: p=0.017) and those on dialysis (3.2% vs. 0.5%: p=0.028) compared to the low Tnl group. Additionally, we did not find any significant difference in 30-day mortality or readmission between the two groups in our cohort. On average, each patient in the high TnI group spent $936 more for hospital admissions compared to patients in the low Tnl group. However, this difference was not statistically significant.

Conclusions

Tnl at admission did not reveal a significant impact on 30-day mortality or readmission, which is consistent with the findings of previous studies. A high Tnl at admission increased the cost of admission; however, the difference was not statistically significant in our cohort.

## Introduction

Acute coronary syndrome (ACS) requires an accurate diagnostic evaluation to improve clinical outcomes. The measurement of cardiac biomarkers has historically been considered a known indicator that prompts escalation to life-saving therapies such as percutaneous coronary intervention (PCI). This is particularly true for troponin levels (Tnl) when used in conjunction with clinical assessment and 12-lead electrocardiogram (ECG) [1]. In recent years, troponin assays have become increasingly beneficial with the introduction of high-sensitivity cardiac troponins (hs-cTn), allowing for improved diagnostic accuracy for acute myocardial infarction (AMI) in patients with chest pain [2]. Additionally, these increasingly sensitive troponins now allow for the detection of minute instances of myocardial necrosis, which has prompted the redefinition of MI among many experts [3]. Despite the rapid evolution of biomarker utility, the degree of troponin elevation at presentation has continually been thought of as a strong and independent predictor of in-hospital mortality and readmission, especially in cases leading to PCI [4]. Recent studies, however, have suggested a lack of clarity regarding this hypothesis. Given that PCI is an increasingly common procedure in the United States (US) with high rates of readmission, this ambiguity is concerning [5,6]. Compelling evidence has even revealed a lack of complete understanding surrounding the biochemical mechanisms of cardiac troponin release, which further challenges these previous notions [7]. Noncardiac causes have been evidenced as common etiologies for worsening outcomes and readmission in those who have received PCI, but initial Tnl has not been linked to these variables [8]. Key studies such as the randomized assessment of treatment using panel Assay of cardiac markers (RATPAC) trial [9] have attempted to link the measurement of cardiac biomarker levels with both hospital outcomes and cost, but the validity of their findings has been challenged since. This further reinforces the element of confusion surrounding troponin and its role in predicting overall hospital costs. Elucidating the association of Tnl at presentation with mortality, readmission rates, and overall hospital costs can potentially help to further determine the value of cardiac biomarkers. In this study, we attempted to analyze the association between a dichotomous troponin cut-off and short-term mortality as well as cost. Higher Tnl has previously been associated with a worse outcome. This study also attempted to address the cost issue.

## Materials and methods

Patient population and study design

We performed a retrospective chart review of 7,389 patients who underwent coronary angiogram at OSF Saint Francis Medical Center between 2015 and 2017. Institutional Review Board approval was obtained from the office of Human Research at the University of Illinois Chicago at Peoria, IL. Considering the retrospective nature of this study, a consent waiver was granted. 

The initial sample size was 7,389. Patients with incomplete records, those without initial troponin values, and those who required coronary artery bypass grafting (CABG) were excluded. After excluding patients who were deemed ineligible, the final sample size of 1,163 was reached.

Clinical data were extracted retrospectively, and all patients had their baseline ECG and Tnl obtained before undergoing coronary angiogram. Clinical variables studied included age, gender, body mass index (BMI), smoking history, history of hypertension, diabetes, family history of coronary artery disease (CAD), prior MI, prior heart failure, valve surgery, prior PCI, prior CABG, prior cerebrovascular disease, prior peripheral arterial disease (PAD), chronic lung disease, prior cardiac shock, prior cardiac arrest, and renal disease requiring dialysis.

Patients were divided into two groups depending on their Tnl at presentation; a cut-off of 0.04 ng/ml was used based on the laboratory reference values. Patients with Tnl of <0.04 ng/ml were called the low TnI group and patients with Tnl of ≥0.04 ng/ml were placed in the high TnI group.

Outcome comparison

Primary outcomes were 30-day all-cause readmission and all-cause mortality. The secondary outcome was the admission cost.

Statistical analysis

Baseline characteristics and clinical data were compared between the groups. Continuous data were represented as mean ± standard deviation (SD) and categorical data as proportions and percentages. A t-test was used to compare continuous variables and the chi-square test for categorical variables. Adjusted statistical analyses were conducted to compare clinical variables. A multivariable analysis was performed to evaluate clinical predictors of 30-day readmission, 30-day mortality, and cost. All calculations were performed using Stata software, v12 (StataCorp LLC, College Station, TX), and a p-value of less than 0.05 was considered statistically significant.

## Results

Sample demographics

Among a total of 1,163 patients, there were 207 patients in the low TnI group and 956 patients in the high TnI group (Table 1). The mean age was 63.1 (SD: 11.3) vs. 64.9 (SD: 13.0) years for patients in the low and high TnI groups, respectively. The low TnI group had a significantly higher percentage of male patients compared to the high TnI group (76.3 vs. 69.5; p=0.049).

**Table 1:** Baseline demographics *Chi-square test for categorical variables and t-test for continuous variables Tnl: troponin level; SD: standard deviation; BMI: body mass index; CAD: coronary artery disease; MI: myocardial Infarction; HF: heart failure; PCI: percutaneous coronary intervention; CABG: coronary artery bypass grafting; CVD: cardiovascular disease; PAD: peripheral arterial disease; CCS: Canadian Cardiovascular Society; NSTEMI: non-ST-elevation myocardial infarction

Continuous variables	Total (n=1,163), mean (SD)	Tnl of <0.04 (n=207), mean (SD)	Tnl of ≥0.04 (n=956), mean (SD)	P-value*
Age	64.6 (12.7)	63.1 (11.3)	64.9 (13.0)	0.076
BMI	30.5 (6.8)	30.6 (6.5)	30.5 (6.8)	0.902
Categorical variables	All (n=1,163), n (%)	Tnl of <0.04 (n=207), n (%)	Tnl of ≥0.04 (n=956), n (%)	P-value*
Male	822 (70.7)	158 (76.3)	664 (69.5)	0.049
Smoker	400 (34.4)	81 (39.1)	319 (33.4)	0.114
Hypertension	859 (73.8)	155 (74.9)	704 (73.6)	0.713
Family history of CAD	148 (12.7)	16 (7.7)	132 (13.8)	0.017
Prior MI	269 (23.1)	68 (32.8)	201 (21.0)	<0.001
Prior HF	130 (11.2)	18 (8.7)	112 (11.7)	0.211
Valve surgery	19 (1.6)	3 (1.4)	16 (1.7)	0.817
Prior PCI	308 (26.5)	74 (35.7)	234 (24.5)	0.001
Prior CABG	96 (8.2)	18 (8.7)	78 (8.2)	0.799
Current dialysis	32 (2.7)	1 (0.5)	31 (3.2)	0.028
Prior CVD	168 (14.4)	32 (15.5)	136 (14.2)	0.647
Prior PAD	136 (11.7)	32 (15.5)	104 (10.9)	0.063
Chronic lung disease	188 (16.2)	34 (16.4)	154 (16.1)	0.911
Diabetes	387 (33.3)	66 (31.9)	321 (33.6)	0.639
Prior cardiac shock	27 (2.3)	3 (1.4)	24 (2.5)	0.358
Prior cardiac arrest	50 (4.3)	8 (3.8)	42 (4.4)	0.734
CAD at presentation				<0.001
1: No symptoms	17 (1.4)	1 (0.5)	16 (1.7)	
2: Atypical symptoms	12 (1.0)	3 (1.4)	9 (0.9)	
3: Stable angina	25 (2.1)	9 (4.3)	16 (1.7)	
4: Unstable angina	172 (14.8)	104 (50.2)	68 (7.1)	
5: NSTEMI	691 (59.4)	27 (13.0)	664 (69.5)	
6: STEMI	246 (21.1)	63 (30.4)	183 (19.1)	
Angina class				0.003
1: No symptoms	45 (3.9)	5 (2.4)	40 (4.2)	
2: CCS I	14 (1.2)	3 (1.4)	11 (1.1)	
3: CCS II	80 (6.9)	20 (9.7)	60 (6.3)	
4: CCS III	164 (14.1)	44 (21.3)	120 (12.5)	
5: CCS IV	860 (73.9)	135 (65.2)	725 (75.8)	
Received PCI				0.116
No PCI	59 (5.07)	6 (2.9)	53 (5.54)	
PCI	1,104 (94.93)	201 (97.1)	903 (94.46)	

Clinical variables and troponin levels

**Figure 1:**
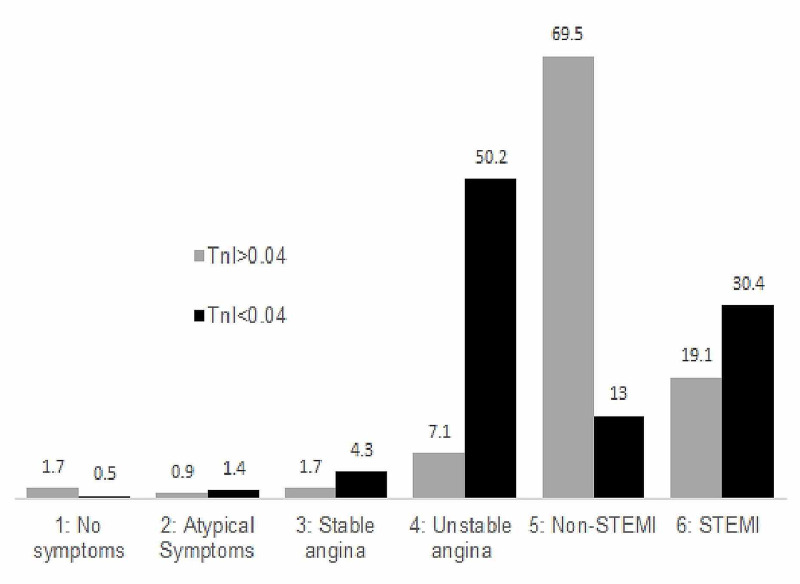
Troponin level vs. CAD at presentation Percentage bar graphs comparing CAD at presentation vs. troponin levels Tnl: troponin level; CAD: coronary artery disease; STEMI: ST-elevation myocardial infarction

A significant number of patients in the high TnI group reported a positive family history of CAD (13.8% vs. 7.7%; p=0.017) and were more likely to be on dialysis (3.2% vs. 0.5%; p=0.028) compared to the low Tnl group. However, the high TnI group had a significantly lower number of patients with previous MI (21% vs. 32.8%; p: <0.001) and history of previous PCI (24.5% vs. 35.7%; p=0.001). Based on the initial clinical picture, a higher number of patients with high TnI were found in the non-ST-elevation MI group (NSTEMI) category (69.5%; p=0.001) compared with the unstable angina category (50.2%; p=0.001) (Figure 1). A majority of patients in both groups underwent PCI (high TnI: 94.46%; low TnI: 97.1%); however, the difference was not statistically significant.

Predictors of elevated troponin levels

On linear regression analysis (Table 2), patients with a significant family history of CAD had 2.68 times higher odds of being in the high TnI group [odds ratio (OR): 2.68, 95% CI: 1.37-5.27, p=0.004]. Similarly, patients on dialysis were 9.1 times more likely to be in the high TnI group compared with patients not on dialysis (OR: 9.12, 95% CI: 1.12-74.01, p=0.038). Patients who presented with stable (OR: 0.07, 95% CI: 0.01-0.85, p=0.037) and unstable angina (OR: 0.03, 95% CI: 0.00-0.35, p=0.005) had significantly lower odds of being in the high TnI group.

**Table 2:** Linear regression analysis for clinical variables in patients with elevated troponins BMI: body mass index; CAD: coronary artery disease; MI: myocardial Infarction; HF: heart failure; PCI: percutaneous coronary intervention; CABG: coronary artery bypass grafting; CVD: cardiovascular disease; PAD: peripheral arterial disease

Covariates	Odds ratio	P-value	95% CI	
Age	1.01	0.480	0.99	1.03
BMI	1.00	0.927	0.97	1.03
Male	0.82	0.354	0.53	1.26
Smoker	0.94	0.771	0.61	1.45
Hypertension	0.86	0.528	0.55	1.36
Family history of CAD	2.68	0.004	1.37	5.27
Prior MI	0.75	0.333	0.43	1.34
Prior HF	1.80	0.085	0.92	3.50
Valve surgery	0.89	0.887	0.18	4.47
Prior PCI	0.76	0.328	0.43	1.32
Prior CABG	1.25	0.554	0.60	2.59
Current dialysis	9.12	0.038	1.12	74.01
Prior CVD	1.24	0.447	0.71	2.16
Prior PAD	0.58	0.072	0.32	1.05
Chronic lung disease	0.83	0.471	0.49	1.39
Diabetes	1.25	0.317	0.81	1.92
Prior cardiac shock	1.53	0.538	0.40	5.92
Prior cardiac arrest	1.74	0.219	0.72	4.23

Troponin level vs. 30-day readmission, 30-day mortality, and cost

**Figure 2:**
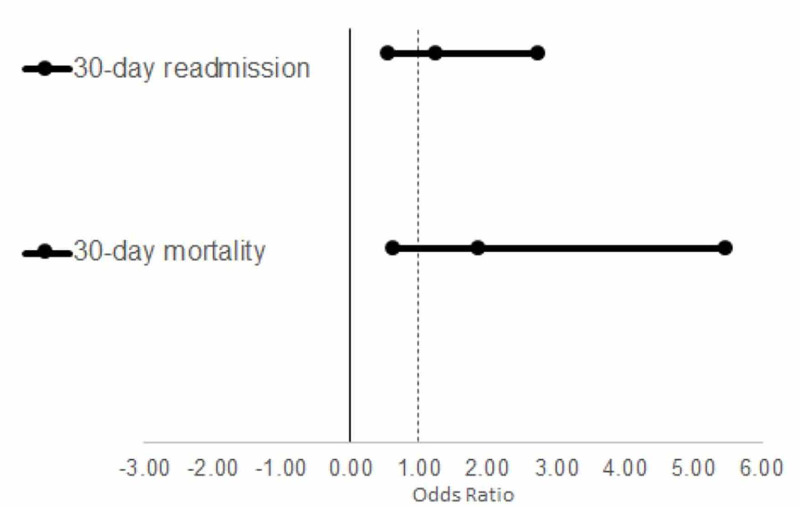
Odds ratio of 30-day readmission and mortality in patients with elevated troponins

**Figure 3:**
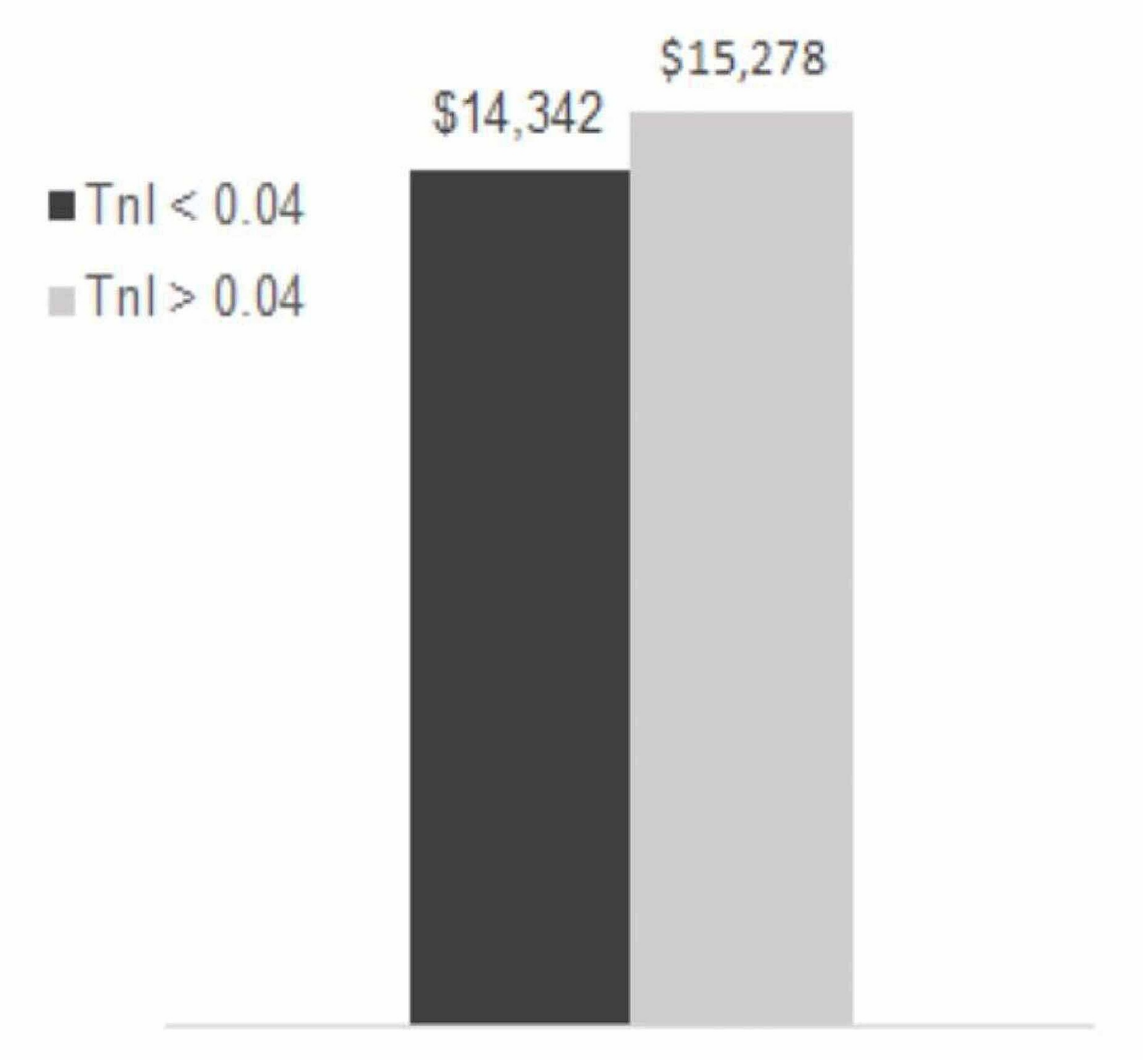
Troponin level vs. cost Tnl: troponin level

Although patients in the high TnI group showed increased odds of 30-day readmission (OR: 1.24, 95% CI: 0.56-2.74) and 30-day mortality (OR: 1.87, 95% CI: 0.64-5.48), these observations did not reach statistical significance (p=0.593 and p=0.254, respectively) (Figure 2). The high TnI group demonstrated a slight increase in cost for their initial care ($15,278 vs. $14,342) (Table 3); however, this also did not reach statistical significance (p=0.261) (Figure 3).

**Table 3:** Multivariate analysis comparing cost in low and high troponin groups, with different CAD presentation types and stages of angina Tnl: troponin level; CAD: coronary artery disease;* *CCS: Canadian Cardiovascular Society; NSTEMI: non-ST-elevation myocardial infarction

Sample	Tnl of <0.04	Tnl of ≥0.04	Difference	95% CI		P-value
All	$14,342	$15,278	$936	-$697	$2,570	0.261
CAD at presentation						
3: Stable angina	$8,092	$11,550	$3,458	-$5,571	$12,487	0.453
4: Unstable angina	$11,981	$10,997	-$984	-$3,427	$1,459	0.430
5: NSTEMI	$12,209	$14,441	$2,233	-$1,104	$5,569	0.190
6: STEMI	$18,463	$18,798	$335	-$3,697	$4,367	0.871
Angina class						
1: No symptoms	$22,109	$33,495	$11,386	-$7,162	$29,935	0.229
3: CCS II	$11,831	$10,864	-$967	-$4,457	$2,523	0.587
4: CCS III	$10,834	$14,503	$3,670	$52	$7,288	0.047
5: CCS IV	$15,153	$14,802	-$351	-$2,308	$1,605	0.725

## Discussion

Summary of findings

Our study involved an initial sample of 1,163 patients undergoing an angiogram. We divided the patients into two groups based on Tnl at presentation with a cut-off value of 0.04ng/ml. We found a significant association of family history of CAD and end-stage renal disease (ESRD) with patients presenting with high Tnl. This was confirmed on logistic regression analysis. In general, patients with STEMI and NSTEMI constituted the majority in the high Tnl group (Figure 1).

Although the 30-day all-cause readmission and 30-day all-cause mortality were higher in the high Tnl group, they did not reach statistical significance (Figure 2). While the high Tnl group incurred a higher cost of admission ($15,278$ vs.14,342), this relationship did not reach statistical significance either (p=0.261) (Figure 3).

Literature review

It is well established that elevated Tnl is a key component in early risk stratification and management of ACS [1]. Similarly, the application of risk scores incorporating Tnl in the management of these patients is associated with improved patient outcomes, mostly from early and aggressive treatment [1].

In this retrospective analysis of consecutive patients undergoing angiogram, we evaluated the association of Tnl at initial presentation with short-term outcomes of overall 30-day all-cause mortality, hospital readmission rates, and admission cost.

There had been attempts to clarify the utility of cardiac-specific troponins (TnI and TnT) with respect to the cost of care. Initial studies comparing creatinine phosphokinase-MB with troponin-T showed a cost-benefit in favor of Troponin T, with shorter lengths of stay and hospital cost in suspected acute MI patients [10]. In some studies, however, the use of troponin had reportedly led to an increase in the cost of care, out of proportion to the degree of benefits. In a 2016 study of 56,895 inpatient admissions, initial troponin testing alone increased the cost by 22.6%. An elevated troponin raised the cost further by 74.2% regardless of a diagnosis of AMI due to the greater use of ancillary testing. This is potentially either due to false detection or detection due to reasons other than AMI [11].

Our study was an attempt to look into the predictive power of troponin pertaining to hospital outcomes and cost comparison for admission.

Interpretation of results

Despite the well-known fact that cardiac troponins correlate with infarct size, the RUTI-STEMI study failed to establish the prognostic utility of troponin elevation (both cTn and hs-CTn) in predicting clinical outcomes in the short and long term in the setting of STEMI (30-day and 30-day to one-year periods) [12]. Our study did not find a prognostic relationship between elevated Tnl and short-term outcomes in patients undergoing angiography as well.

The results of the study need to be interpreted in light of several factors. First of all, the timing of the biomarker release into the circulation is dependent on the blood flow and timing of blood draw with the onset of symptoms. As biomarker release is substantially dependent on blood flow, there is significant variability in the time to reach a peak value (velocity), the time when a normal value may become greater than the 99th percentile, or when a change of pattern can be observed [3]. Therefore, a negative level may not reflect the true acuity of disease and vice versa; the same can be projected for the clinical outcomes and cost [13].

Although the study results are neutral, these have a greater implication in the current day practice as there is much concern about the overutilization of resources in the healthcare system. Further studies aiming at an analysis of cost utilization with respect to Tnl will shed more light to pave the way for practice-changing recommendations, which will eventually lead to proper resource utilization and avoiding unnecessary over-testing.

Limitations

This study was a retrospective analysis, which has its inherent limitations. Regarding troponin-I levels, it is noteworthy that the levels may be normal in some patients based on temporal correlation with symptom onset.

Unlike a peak troponin (based on a rise and a fall), an isolated Tnl mostly underestimates the degree of injury as it could take 6-36 hours for it to peak and seldom represents the continuum. Our study looked only at the initial Tnl. The peak Tnl was studied mostly in the setting of STEMI, but data were missing in the setting of NSTEMI and unstable angina. As the troponin value mentioned is only the initial one, it is noteworthy that the diagnosis of STEMI may have a normal range value, and the diagnosis of unstable angina may have a positive value, depending upon the time when it was drawn.

Although it is well known that an elevated troponin in dialysis patients does independently predict poor outcomes, due to the peculiar elimination kinetics (renal reticuloendothelial system), patients with ESRD have an elevated baseline troponin. Hence, high levels in these patients may not represent true cardiac events.

## Conclusions

We did not find any significant association between initial Tnl in patients undergoing cardiac angiogram and short-term outcomes including 30-day all-cause readmission and all-cause mortality. A high Tnl at presentation was associated with an increase in the cost of admission; however, the difference was not statistically significant.
